# The COVID-19 pandemic had heterogeneous effects on migration dynamics

**DOI:** 10.1073/pnas.2529730122

**Published:** 2025-12-15

**Authors:** Jordan D. Klein, Ingmar Weber

**Affiliations:** ^a^Department of Digital and Computational Demography, Max Planck Institute for Demographic Research, Rostock 18057, Germany; ^b^Department of Computer Science, Saarland University, Saarbrücken 66123, Germany

Chi et al. (1) make an important contribution to the international migration literature by using digital trace data to estimate monthly global bilateral migration flows for 2019–2022 and, by releasing these data publicly, provide an invaluable resource.

Because their data span the COVID-19 pandemic, the authors are uniquely positioned to examine how it reshaped global migration dynamics. They report that “global migration flows fell 64% in 2020, driven in part by the imposition of border controls,” and that “immigration is negatively correlated with the COVID-19 policy stringency index” (1).

Their findings, that overall migration flows decreased during the pandemic and that more stringent policies were associated with decreased inflows, align with previous literature (2–7). While the authors’ conclusions capture the broad declines in cross-border movement observed during the pandemic’s first year, conflating the impacts of border closures, broader sets of disease-control policies, and exogenous effects of the pandemic itself risks obscuring the directionally heterogeneous effects that different policies had on international migration. Contextualizing how general restrictions on activity and mobility primarily reduced inflows, whereas border closures mainly restricted outflows, is essential for interpreting these results and designing more effective policy responses to future epidemics and pandemics.

The COVID-19 policy stringency index referenced by the authors aggregates nine categories of disease-control policies (8), including a range of domestic measures and restrictions on international travel. Using digital-trace-derived estimates of month-to-month changes in migrant stocks, Klein, Weber, and Zagheni (2) found that greater stringency of domestic restrictions not related to international travel was associated with decreases in migrant stocks in countries implementing such policies, indicating that reduced inflows dominated, a finding in line with those of Chi et al. (1). However, when controlling for this broader suite of policies, pandemic-related health impacts, and the onset of the crisis itself, Klein, Weber, and Zagheni found that travel bans corresponded to increases in migrant stocks from affected countries, suggesting that curtailed outflows were the prevailing mechanism. This interpretation is consistent with contemporaneous analyses by Benton et al. (6), who observed that travel restrictions and border closures stranded large numbers of regular migrants and travelers, who faced high financial and logistical barriers to repatriation and return migration. Simultaneously, fewer displaced people were able to return to their countries of origin. Border closures may indeed have reduced total flows, as Chi et al. (1) imply, but not necessarily in the direction one might expect-policymakers should consider these potential unintended consequences.

Taken together, these findings point to a need for greater attention to the directional heterogeneity of migration dynamics during the COVID-19 pandemic. Focusing on total flows alone obscures the distinct mechanisms through which different policy instruments influence in- and out-migration, including potential unintended or paradoxical effects that policymakers should account for when planning future disease response. Chi et al.’s (1) dataset offers precisely the temporal and directional resolution needed to disentangle these mechanisms. We commend the authors for releasing this resource and encourage future work using it to unpack these directional dynamics and the distinct policy pathways through which they are produced.
